# Evidence for preservation of vacuolar compartments during foehn-induced chalky ring formation of *Oryza sativa* L.

**DOI:** 10.1007/s00425-018-2975-x

**Published:** 2018-08-11

**Authors:** Yuto Hatakeyama, Chisato Masumoto-Kubo, Hiroshi Nonami, Satoshi Morita, Kenzo Hiraoka, Yayoi Onda, Taiken Nakashima, Hiroshi Nakano, Hiroshi Wada

**Affiliations:** 1 Kyushu Okinawa Agricultural Research Center, National Agriculture and Food Research Organization, Chikugo, 833-0041 Japan; 2 Graduate School of Agriculture, Ehime University, Matsuyama, 790-8566 Japan; 3 Clean Energy Research Center, The University of Yamanashi, Kofu, 400-8511 Japan; 4 Research Faculty of Agriculture, Hokkaido University, Sapporo, 060-8589 Japan

**Keywords:** Low water potential, Milky-white rice, Osmotic adjustment, Starch, Vacuole

## Abstract

Vacuolar compartments being sustained among the amyloplasts inadequately accumulated in rice endosperm cells are the main cause of chalky ring formation under dry wind conditions.

Foehn-induced dry wind during the grain-filling stage induces shoot water deficit in rice (*Oryza sativa* L.) plants, which form a ring-shaped chalkiness in their endosperm that degrades milling quality and rice appearance. Air spaces formed in several inner cells cause significant transparency loss due to irregular light reflection. Although starch synthesis was suggested to be retarded by osmotic adjustment at foehn-induced moderately low water potential, the source of these air spaces remains unknown. We hypothesised that the preservation of vacuoles accompanied by a temporary reduction in starch biosynthesis in the inner cells leads to the chalky ring formation. Panicle water status measurement, light and transmission electron microscopic (TEM) observations, and an absolute qPCR analysis were conducted. Most starch synthesis-related genes exhibited temporarily reduced expression in the inner zone in accordance with the decrease in panicle water status. TEM observations provided evidence that vacuolar compartments remained among the loosely packed starch granules in the inner endosperm cells, where a chalky ring appeared after kernel dehydration. Taken together, we propose that vacuolar compartments sustained among the amyloplasts inadequately accumulated in rice endosperm cells and caused air space formation that leads to ring-shaped chalkiness under dry wind conditions.

**Electronic supplementary material:**

The online version of this article (10.1007/s00425-018-2975-x) contains supplementary material, which is available to authorized users.

## Introduction

Crop production is frequently affected by environmental stresses, particularly at the reproductive stage. In rice (*Oryza sativa* L.), deterioration in the appearance and milling quality due to an increase in chalky rice has long been a serious concern in rice production globally (Hoshikawa 1989; Jagadish et al. 2015; Morita et al. 2016b). A major chalky rice, called milky-white rice (MWR), exhibits a chalky ring over several cell layers in the endosperm, in which starch accumulation occurring from the centre to the periphery of the kernel during the early and middle stages of accumulation is inadequate (Juliano 1985; Hoshikawa 1989). Consequently, air spaces are generated among the inadequately accumulated starch granules, which cause irregular light reflection (Tashiro and Wardlaw 1991) and reduce transparency, resulting in a chalky ring appearance in the endosperm. This type of chalkiness has been frequently observed when plants are exposed to several environmental stresses in the ripening stage, such as high temperature (Tashiro and Wardlaw 1991; Morita 2000) and short-term hot and dry wind conditions (Ishihara et al. 2005; Wada et al. 2011). In general, starch metabolism has received most attention in the study of stress-induced chalky formation (Zakaria et al. 2002; Yamakawa et al. 2007; Zhang et al. 2011; Wada et al. 2014). The observed loosely packed starch granules are a prerequisite for chalky formation; however, they may be insufficient to explain the physical cause of the transparency loss confined to part of the kernel. In the cell compartment, what exactly becomes the air spaces remains to be identified.

There is growing evidence that the maintenance of vacuolar/vesicle volume in endosperm cells is closely associated with chalky formation. In the case of white-belly and white-core rice, Li et al. (2014) detected a major quantitative trait locus for genetically formed rice chalkiness and identified it as *VHP1;5*, a member of the vacuolar H^+^-pyrophosphatase (V-PPase) gene family. They attributed air spaces to the small vesicle-like structures accumulated in endosperm cells observed by transmission electron microscopy (TEM) (Li et al. 2014). Xi et al. (2014) have suggested that proteins also play an important role on chalky formation in white-belly rice. Although the role of proteins on the air space formation remains obscure, these studies highlighted that other organelles including protein bodies and vesicle or vacuole-like structures in cytosol may also participate in the air space formation in the chalky cells.

Recently, it has been reported that osmotic adjustment occurred at moderately low water potential under hot and dry wind conditions, resulting in chalky ring formation (Wada et al. 2011, 2014). Starch degradation might be involved in this process, although the prefixed ^13^C analysis at the kernel level has suggested that a partial reduction in starch biosynthesis, rather than starch degradation, temporarily occurred during osmotic adjustment at moderately low water potential (Wada et al. 2014). Given the fact that starch accumulation radially occurs from the centre to the periphery of the endosperm under normal conditions (Juliano 1985; Hoshikawa 1989), it is proposed that a significant spatial difference in starch accumulation rates may exist between inner and outer endosperm at low water potential, although this remains to be examined. In addition, no direct evidence for the source organelle(s) of ring-shaped air space formation has been provided.

In this work, we hypothesised that the preservation of vacuoles in the inner osmotically adjusted cells, in contrast with a spatiotemporal reduction in starch biosynthesis, is responsible for air space formation under dry wind conditions. To test our hypothesis, we analysed the time course of changes in the expression of the target genes in the inner and outer endosperm tissues, corresponding to putative chalk and outer translucent zones, respectively, under dry wind conditions. We used genomic DNA-based absolute quantification (Gambetta et al. 2013; Wada et al. 2017) to exclude possible variation in expression of the reference gene(s) that potentially exist under stress conditions. We carried out this analysis and TEM observation at each tissue and found that the observed chalky ring originated from the vacuole-like structures preserved upon osmotic adjustment under dry wind conditions. The expression of the target genes, which included starch metabolism-related genes, two vacuolar proton pumps, V-PPase and vacuolar H^+^-ATPase (V-ATPase)-encoding genes and vacuolar autophagy-related genes as potential candidates, was analysed.

## Materials and methods

### Plant materials

A growth-chamber experiment was conducted in 2013, in accordance with a previous study (Wada et al. 2014). *Oryza sativa* L. cv. ‘Koshihikari’ seeds were provided from Miyazaki Agricultural Research Institute, Miyazaki, Japan. Plants were grown outdoors in pots until the flowering stage. Ten plants per pot were prepared. All tillers were periodically removed to restrict each plant to its main culm to minimise panicle-to-panicle variation and reduce the shielding effect upon exposure to the dry wind treatment (see below). At 5 days after heading (DAH), the plants were transferred to a growth chamber (22/22 °C), 70/80% relative humidity, 0.79/0.53 kPa vapour pressure deficit and 750 μmol photons m^−2^ s^−1^ photosynthetically active radiation at the plant canopy with a photoperiod of 14 h L:10 h D. The grain growth score, ranging from 0 to 1 according to size and developmental stage (see Fig. 1B in Wada et al. 2014), was monitored through kernel development. When the score of inferior kernels attached to the tertiary pedicels on the fourth to sixth secondary rachis branches (middle panicle position) reached a mean score of 0.87 at 13 DAH, the plants were transferred to another growth chamber (34/34 °C, 50/40% relative humidity, 2.66/3.19 kPa vapour pressure deficit and 750 μmol m^−2^ s^−1^ photosynthetically active radiation) at 1200 h and subjected to 48-h dry wind treatments, so that a high frequency of ring-shaped chalky rice could be observed (typically ≥ 30%; see Wada et al. 2014). Wind speed was set at approximately 7 m s^−1^ and directed towards the plant canopy. Other potted plants were kept in the same chamber in a cool and non-dry wind area, and these served as the control. Wind speed at the canopy in the control treatment was 0.2 m s^−1^. After the dry wind experiment, plants were transferred to the control chamber to grow until 30 DAH. Thereafter, the plants were placed outdoors until 40 DAH (maturing stage). Plants were supplied with water daily. For all qPCR assays, inferior spikelets attached to the tertiary pedicels on the middle panicle position were used because they exhibited the highest frequency of ring-shaped chalkiness under dry wind conditions at that stage of development (Wada et al. 2011).

### Plant water status

Panicle water potential (PWP) was determined with a pressure chamber in accordance with the work of Turner (1988) and Wada et al. (2011).

### Microscopy

Transverse sections of the harvested inferior kernels attached to the middle position in a panicle (see above) were photographed at 50× using a digital microscope (KH-3000; Hirox, Tokyo, Japan). Kernel samples for microscopic observation were sampled in growth chambers, fixed and embedded in accordance with a previously reported procedure (Saito et al. 2010, 2012) with slight modifications. Transverse segments (1–2 mm thick) from the middle of the kernel at 13 and 40 DAH were fixed with 4% (w/v) paraformaldehyde in 100 mM sodium phosphate (pH 7.2) for 3 h at room temperature and then washed in 100 mM phosphate buffer (pH 7.2). Fixed tissues were dehydrated through an ethanol series, and embedded in LR white resin in the ‘hard’ formulation (London Resin, Hampshire, UK) by 2-day polymerisation at 60 °C. Semi-thin sections (approximately 900 nm) for light microscopy were stained with 0.1% (w/v) Coomassie Brilliant Blue for 1 h followed by potassium iodide for 1 min, and ultra-thin sections (approximately 80–100 nm) for electron microscopy were stained with lead citrate for 15 min. After the staining, ultra-thin sections were observed with a TEM (JEM-1010, JEOL Ltd., Tokyo, Japan). Sections were cut with an ultramicrotome (Sorvall MT-5000; DuPont, Newtown, CT, USA) using a diamond knife. For the organelle arrangement image analysis, the outlines of all amyloplasts, protein bodies, other area (referred to as *gap*) in the cells, and the cells on the light microscopic images were traced using ImageJ software (U.S. National Institutes of Health, Bethesda, MD, USA).

### Gene expression analysis

The developing kernels were immediately frozen in liquid nitrogen at the time of collection and stored at – 80 °C until further preparation. Tissue segments were carefully extracted using a 2.0-mm skin-biopsy punch (BPP-20F; Kai Industries, Tokyo, Japan) from the middle zone of the frozen kernels along the direction of the short axis of an ellipsoid (Fig. S1). Tissue was extracted on dry ice under a digital microscope (KH-3000; Hirox, Tokyo, Japan). Then, two outer tissue segments, which become translucent at maturation, averaging between 0 and 220 μm below the epidermis (*n *= 99), were removed with a razor blade and pooled; these are referred to as ‘outer tissue’ samples (Fig. S1). The residual column-shaped tissue, having a mean length of 1420 μm (*n *= 99) at the central point, where chalkiness is presumed to form later, is referred to as ‘inner tissue’ samples (Fig. S1).

Gene expression was analysed using genomic DNA-based absolute quantification, in accordance with the work of Gambetta et al. (2013), which is absolute expression normalised to the absolute level of reference gene expression. This method eliminates errors ascribed to variability in reference gene expression among samples and can be used as a proxy for absolute expression, which is needed to produce individual standard curves for every gene of interest. Total RNA from each tissue from three to four kernels per panicle was extracted using RNAs-ici!-S (Rizo Inc., Tsukuba, Japan). The isolated RNA was treated with TURBO DNase (Thermo Fisher Scientific, Waltham, MA, USA), precipitated with ethachinmate (Nippon Gene, Tokyo, Japan) and diluted in DEPC water. RNA of 1 μg was reverse-transcribed using an iScript Advanced cDNA Synthesis kit (Bio-Rad, Hercules, CA, USA) in a total volume of 20 μL. Genomic DNA was prepared from rice caryopses using ISOPLANT (Nippon Gene). Extracted DNA was incubated with RNase (final concentration of 10 μg mL^−1^) at 37 °C for 30 min, precipitated with ethachinmate and resuspended in water.

Quantitative PCR was carried out in a real-time PCR system (CFX 96 Connect; Bio-Rad). Each reaction (5 μL) contained 300 nM of each primer, 0.5 μL of 1:20 diluted cDNA and 2.5 μL of SsoAdvanced SYBR Green Supermix (Bio-Rad). The thermal cycling conditions were first 98 °C for 3 min, followed by 95 °C for 5 s and 60 °C for 10 s for 45 cycles. For amplification of ɑ-amylase 3A (*Amy3A*), autophagy-related genes (*ATG4b* and *ATG7*), cell wall invertase (*CIN2* and *CIN7*), neutral/alkaline invertase (*NIN8)* and sucrose transporter 2 (*SUT2*), at 1:5 diluted cDNA, were used. Dissociation curves for each amplicon were then analysed to verify the specificity of each amplification reaction; the dissociation curves were obtained by heating the amplicon from 65 to 95 °C. No evidence of any primer dimer or formation of other non-specific product was detected for any of the primer pairs used. Each PCR was run in duplicate or triplicate within the same plate, and the C_T_ values obtained from the technical replicates were averaged. To produce a standard curve of *eEF1ɑ*-*1* as a reference gene, the genomic DNA was diluted to obtain a series at 1 log_10_ intervals. The genomic DNA concentration equivalent to the copy number was calculated using the number of base pairs and molecular weight of the *Oryza sativa* haploid genome of 388.82 Mb and 2.57 × 10^11^ mol^−1^, respectively. The copy number of *eEF1ɑ*-*1* of each sample was determined using standard curves (copies of genomic DNA vs. C_T_). The fold of target gene expression to the reference gene from delta *C*_T_ was calculated, and then copies of target genes were determined by fold and copies of *eEF1ɑ*-*1*. Primer sequences used for the amplification are listed in Table S1.

### Kernel quality and weight

Spikelets in each portion in a panicle were further classified in accordance with the work of Matsuba (1991). The appearance of dehulled inferior grains attached to the middle position in a panicle (see above) was objectively evaluated with more than 1.8 mm thickness of grains using a chalky rice grain predictor (RN-850; Kett Ltd., Tokyo, Japan) that is capable for determining the number of MWR kernels by analysis of scanned images of sliced mature kernels (Morita et al. 2016a). Dry weight of kernel samples was determined at harvest.

### Statistical analysis

Analysis of all data was performed using either Student’s *t* test or Tukey–Kramer test in JMP (version 12.1.0; SAS Institute Inc., Cary, NC, USA).

## Results

### Rice appearance and panicle water status

**Fig. 1:**
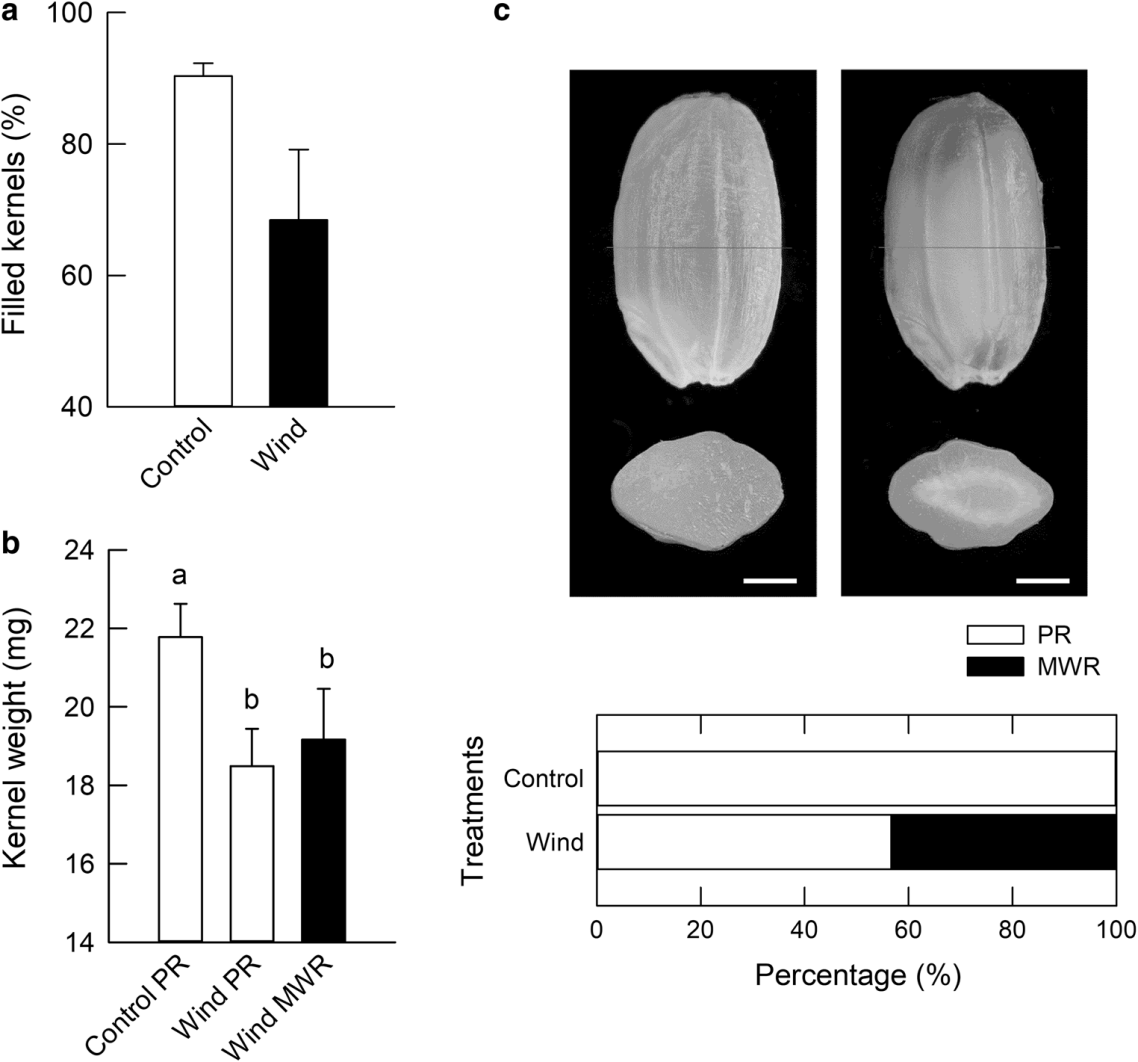
Percentage of filled kernels (**a**), final kernel weight of each group (**b**), and rice appearance (**c**) of inferior grains attached to the tertiary pedicels on the middle position of a panicle grown under 48 h dry wind treatment in growth chambers. In **c**, standing and transverse section images of perfect rice (left) and milky-white rice (right) and increased by applying 48 h dry wind treatment. Note that a ring-shaped chalkiness is indicated in milky-white rice. Each bar indicates 1 mm. Each value in **a** and **b** is the mean ± SE of four panicles and 8–33 kernels from at least three different plants, respectively. Different letters in **b** indicate a significant difference (Tukey–Kramer test, *P* < 0.05). For appearance of each kernel in **c**, significant difference at *P* <0.01 was observed between treatments

**Fig. 2:**
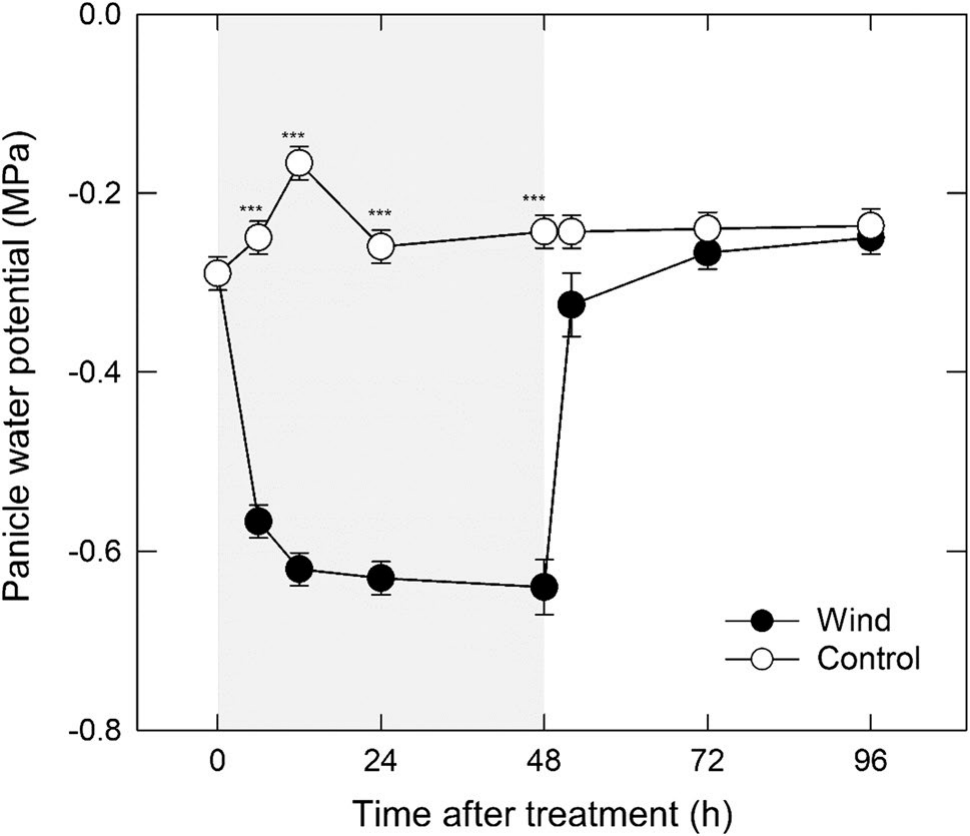
Changes in panicle water potential under 48 h dry wind conditions. Each point is the mean ± SE of 3–4 samples from different plants. Grey areas indicate the 48-h dry wind treatment. ***P < 0.001 by *t* test

Upon exposure to 48-h dry wind treatment, filled kernels and final kernel weight of both perfect rice and MWR declined (Fig. 1a, b), suggesting that dry wind treatment partially inhibited endosperm cell expansion during the growth period, regardless of the kernel type. The proportion of MWR increased in the dry wind treatment up to approximately 43%, resulting in a corresponding decrease in perfect (translucent) rice (Fig. 1c). After subjecting the plants to dry wind treatment, PWP decreased to approximately − 0.63 MPa at 12 h and remained constant throughout the dry wind treatment with no diurnal change (Fig. 2). Later, the dry wind treatment was stopped at 48 h, PWP increased and recovered on the following day to the control level (Fig. 2).

### Expression pattern of starch synthesis-related genes under dry wind conditions

**Fig. 3:**
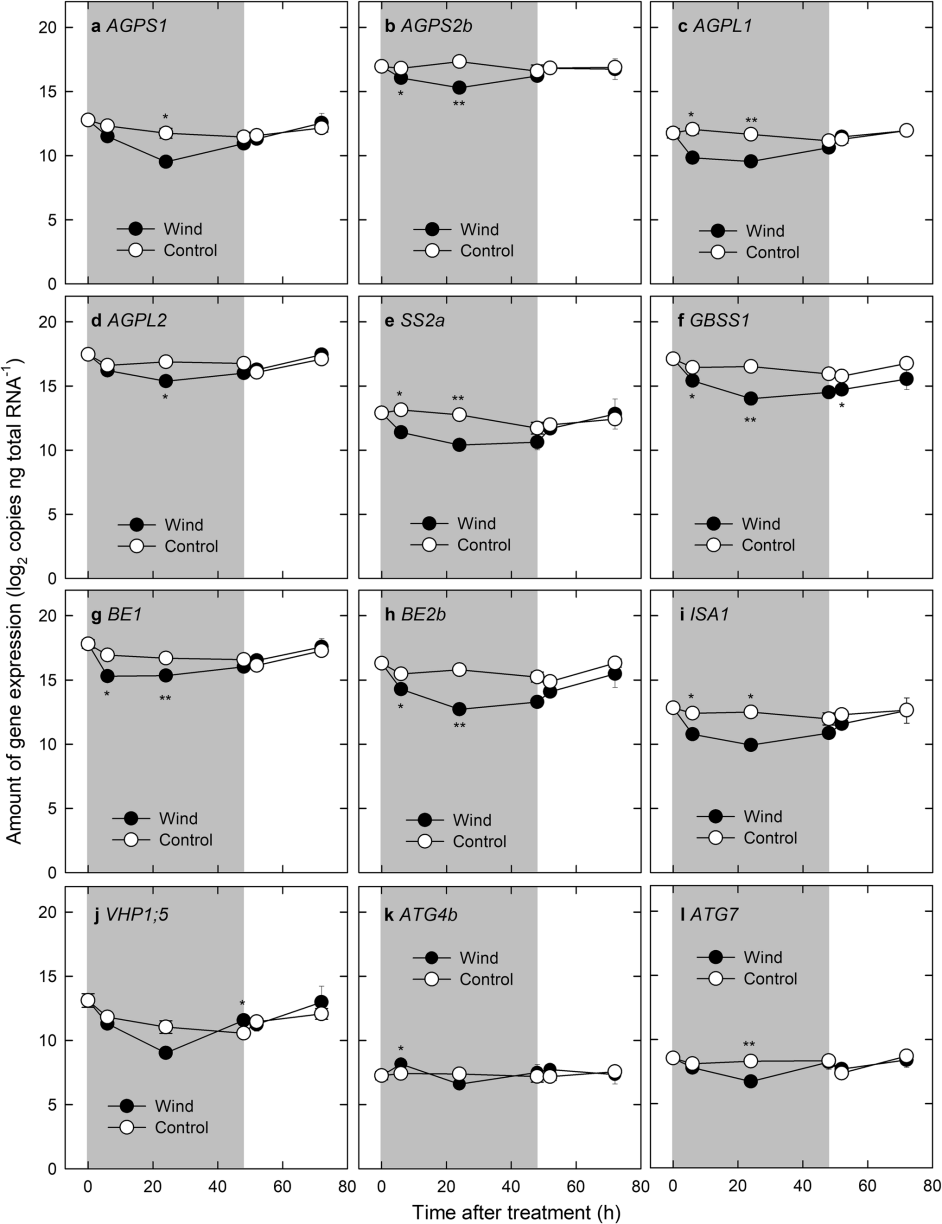
Time course of changes in transcript levels of nine starch synthesis-related genes, vacuolar H^+^-pyrophosphatase gene *(Chalk5)*, and two autophagy-related genes in the inner zone of developing endosperms after dry wind treatment. **a** *AGPS1* (ADP-glucose pyrophosphorylase small subunit 1). **b** *AGPS2b* (ADP-glucose pyrophosphorylase small subunit 2b). **c** *AGPL1* (ADP-glucose pyrophosphorylase large subunit 1). **d** *AGPL2* (ADP-glucose pyrophosphorylase large subunit 2). **e** *SS2a* (starch synthase 2a). **f** *GBSS1* (granule-bound starch synthase 1). **g** *BE1* (branching enzyme 1). **h** *BE2b* (branching enzyme 2b). **i** *ISA1* (isoamylase 1). **j** *VHP1;5* (vacuolar H^+^-pyrophosphatase 1;5). **k** *ATG4b* (autophagy-related gene 4b). **l** *ATG7* (autophagy-related gene 7). Grey areas indicate the 48-h dry wind treatment. Each point is the mean ± SE of 3–4 samples from different plants. Significance at the 0.01 and 0.05 probability levels is indicated by ** and *, respectively

We examined (i) whether starch biosynthesis in the inner endosperm occurs more actively, compared with that in the outer zone in the middle grain-filling stage, and (ii) whether a temporary reduction in starch biosynthesis site specifically occurs in the inner zone, where chalkiness appeared after kernel dehydration. The expression of starch synthesis-related genes was shown to be abundant, exhibiting over 10 log_2_ copies per ng total RNA, consistent with the starch accumulation pattern at the middle grain-filling stage (see Discussion). Conversely, the expression of *Amy3A*, known to be upregulated upon exposure to long-term (at least 10 days) high temperature (Hakata et al. 2012), was shown to be negligible (Table S2). Proteins encoded by other members of the *Amy* gene family, including *Amy1A*, *Amy1C*, *Amy2A*, *Amy3D*, and *Amy3E*, were not detected in the inner endosperm using the same genomic DNA-based absolute quantification (data not shown). Even with some diurnal oscillation observed in the control, the expression of major starch biosynthesis-related genes, such as *AGPS2b*, *AGPL2*, *GBSS1*, and *BE1*, was consistently downregulated under dry wind treatment and reached a minimum level in 24 h (Fig. 3a–i and Table S2). After stopping the dry wind treatment at 48 h, the expression of most genes began to be upregulated, reaching the level of the control at the following day (see 72 h in Fig. 3a–i), in accordance with PWP recovery (Fig. 2). *GBSS1* expression slowly recovered, remaining at a slightly lower level than in the control group after stopping dry wind treatment (Fig. 3f). There was a transient increase at 6 h for the expression of *SS3a*, which thereafter maintained a high level (Table S2), regardless of the changes in PWP (Fig. 2). This differed from the expression of other genes. In addition, the reduction in the expression of amyloplast-localised enzyme-encoding genes, *SS2a*, *GBSS1*, *BE2b*, and *ISA1*, occurred much earlier than that of cytosolic AGPase-encoding genes, *AGPS2b*, and *AGPL2,* in response to low water potential (Fig. 3a–c).

The excised outer zone of rice caryopsis, which turns translucent, is composed of the outer endosperm and pericarp. The volumetric ratio of pericarp to the entire outer tissue was approximately 12.5% at the stage of development, indicating that the outer endosperm was predominant, and the results analysed with the segments can be regarded as reflecting outer endosperm. In the outer zone of the rice caryopsis, the relative gene expression of major starch biosynthesis-related 10 genes (*AGPS1*, *AGPS2b*, *AGPL1*, *AGPL2*, *SS2a*, *GBSS1*, *BE1*, *BE2b*, *ISA1*, and *PUL*) was temporarily downregulated at low water potential (Table S2). Relative expression of *GBSS1* under dry wind conditions declined severely in the outer zone (Table S2).

### Expression pattern of other tested genes under dry wind conditions

The expression of genes encoding sugar transporters related to starch synthesis was also suppressed by dry wind treatment in the inner zone (Table S2). The expression of *BT1*-*2* encoding the ADP-glucose transporter localised at the amyloplast membrane was rapidly decreased by dry wind treatment, and severely suppressed at 24 h (Table S2). Dry wind treatment also inhibited gene expression of vacuolar sugar transporters in the inner zone. The expression of *SUT2* and *TMT1*, which encodes a tonoplast-localised sucrose transporter and monosaccharide transporter, respectively, responded rapidly to the dry wind treatment and remained suppressed throughout treatment (Table S2). There was no expression detected for vacuolar invertase (*VIN1*) (not shown). In addition, absolute amounts of gene expression for invertases localised in the cell wall (*CIN2* and *CIN7*) and the cytosol (*NIN8*) were fewer than 100 copies ng total RNA^−1^ in the inner endosperm (Table S2), suggesting that they were all negligible. In the outer zone, expression of *Susy2*, *Susy3*, and *SUT2* was also downregulated at low water potential.

The absolute amount of expression of one of the vacuolar proton pump-related genes, *VHP1;5*, in the inner zone was less than that of the control after 24 h, but with greater than 9 log_2_ copies per ng total RNA. Thereafter, the expression elevated until 48 h (Fig. 3j); however, it was suppressed in the outer zone during the dry wind treatment (Table S2). The expression levels of autophagy-related genes, *ATG4b* and *ATG7*, in the inner zone were greater than those in the outer zone. Dry wind treatment induced a transient increase in *ATG4b* expression at 6 h, although no treatment difference was observed after 6 h, but there were slight daily fluctuations (Fig. 3k). In contrast, temporary downregulation in *ATG7* expression was observed only at 24 h under dry wind conditions (Fig. 3l).

### Spatial rearrangement of organelle compartmentation altered under dry wind treatment

**Fig. 4:**
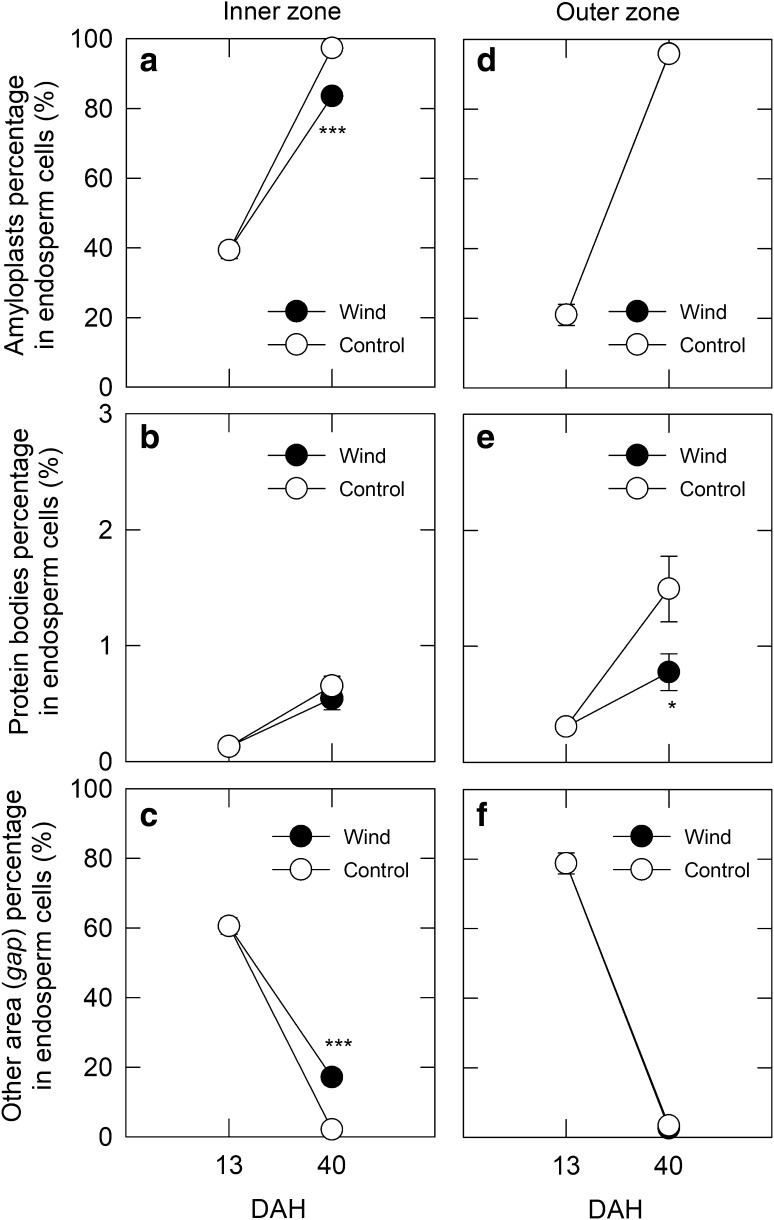
Changes in area-based percentages of major organelles, amyloplasts (**a**, **d**), protein bodies (**c**, **e**), and other area (gap) (**c**, **f**) occupying the inner (**a**–**c**) and outer (**d**–**f**) endosperm cells in each treatment from 13 (initiation of dry wind treatment) to 40 DAH (maturation). Opened and closed circles indicate control and dry wind treatments, respectively. 13 and 40 DAH correspond to the initiation of dry wind treatment and maturation, respectively. Each point with bars is the mean ± SE of 7–10 cells from three different kernels. *** and * show *P* < 0.001 and 0.05 by *t* test, respectively

**Fig. 5:**
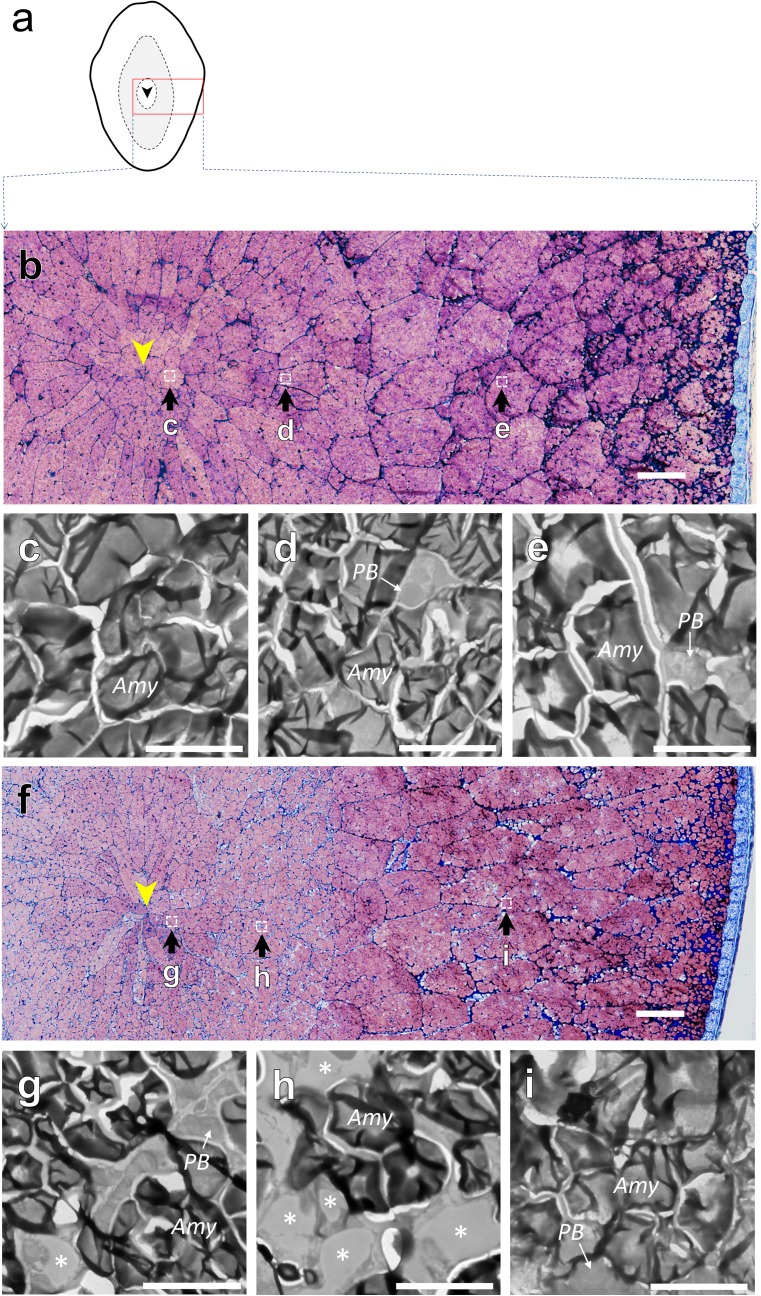
**a** Schematic of transverse section of ring-shaped chalky rice. Images of the transverse sections from central point towards the outer endosperm along with the direction of kernel width in control (**b**) and dry wind treatment (**f**) at 40 DAH, corresponding to the rectangle area shown in red in **a**. Representative TEM images of central (**c**, **g**), middle (both corresponding to the ‘inner’ zone analysed in Fig. 3) (**d**, **h**), and outer (**e**, **i**) endosperm cells in control (**c**–**e**) and dry wind treatment (**g**–**i**), corresponding to the three white squares shown in **b** and **f**. In **a**, **b**, and **f**, each arrowhead indicates the central point of endosperm. In **c**–**e** and **g**–**i**, each *PB*, *Amy*, and asterisk indicates protein body, amyloplast, and vacuolar-like structure remained in the cytosol in **g** and **h**. Bars = 100 μm (**b**, **f**) and 5 μm (**c**–**e**, **g**–**i**)

The image analysis of light microscopy showed that there was a clear difference in organelle compartmentation between the inner and outer zones (Fig. 4). In the inner zone, amyloplast development appeared to precede that of the outer zone (Fig. 4 a, d). In the control, amyloplasts almost completely occupied the cells with minor accumulation of protein bodies (ca. 0.5%), and there was no gap space at maturation (Fig. 4a–c). Dry wind treatment partially inhibited amyloplast development, but with little change in protein bodies, and consequently the other gap area increased, reaching approximately 18% at maturation (Fig. 4a–c). In contrast, there was no difference in amyloplast development and gap formation between treatments in the outer zone at maturation, although the percentage of protein bodies declined in the dry wind treatment. The percentage of protein bodies in the outer zone was greater than that in the inner zone. The percentage of gap space estimated in the first and second cell layers around the central point in control and dry wind treatments was negligibly small, corresponding to 1.46% (*n *= 5) and 1.70% (*n *= 4), respectively. However, when the gap spaces in the transverse section of dry wind-treated kernels (see Fig. 5f) were further inspected by TEM, very few vacuole-like structures were observed in the central endosperm cells under dry wind conditions (Fig. 5g), which are often reported to turn translucent (see Fig. 1c). Starch granules were well packed in the corresponding zone of control endosperms, and there were no similar structures in control kernels (Fig. 5c). In the inner chalky zone of dry wind treatment, where a chalky ring forms, numerous vacuole-like structures appeared to remain among inadequately developed amyloplasts with size heterogeneity in the cytosol (Fig. 5h). In the same zone, storage proteins were also observed to accumulate insufficiently in protein storage vacuoles (Fig. 5h). In contrast, amyloplasts were well packed in the outer endosperm in dry wind treatment, and no vacuole-like structures were found (Fig. 5i), similar to that in control (Fig. 5e). The size of the vacuolar structures observed in the inner zone of dry wind-treated kernels was calculated to be 2.6 ± 1.2 μm (mean ± SD, *n* = 26, 1.0–6.2 μm) in diameter. The range of their volume was estimated to be between 0.5 and 122 fL. When kernel dehydration proceeded in the chalky zone upon dry wind treatment, tonoplasts appeared to be degraded in those cells (Fig. S2).

## Discussion

**Fig. 6:**
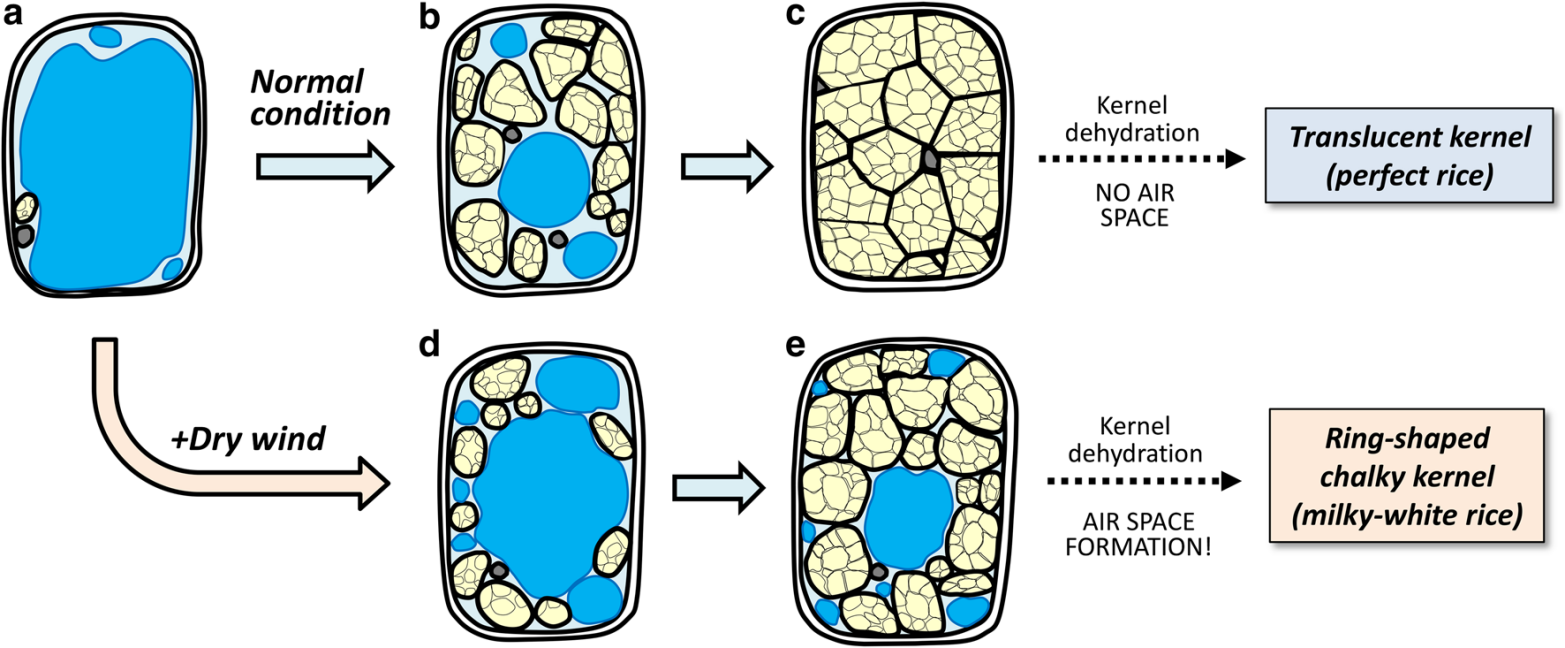
Schematic diagrams describing the putative process of chalky formation in the inner cells of developing kernels exposed at moderately low water potential under dry wind conditions. In each panel, vacuole-like components are indicated in blue. Amyloplasts and protein bodies are indicated in yellow and grey organelles, respectively. To simplify schematics, other organelles, such as nucleus and mitochondria, have been removed. In the inner cells under non-stress conditions (control), cell expansion, accompanied with vacuole enlargement, occurred first, followed by starch accumulation in the cells (see text) (**a**–**c**). As the packing density of amyloplasts in the cells increases, the volume occupied with vacuole-like organelles would decrease, resulting in adequate starch accumulation (**b**–**c**). The situation could be altered, when osmotic adjustment occurred in the cells at moderately low water potential caused by dry wind. In addition to the spatiotemporal reduction in starch biosynthesis, vacuolar structures could have remained in the cells due to osmotic adjustment (**a**–**d** and **d**–**e**), resulting in air space formation in the cells during kernel dehydration

In this work, we hypothesised that vacuoles may remain in the inner endosperm cell, specifically as a consequence of osmotic adjustment under hot and dry wind conditions, resulting in a ring-shaped chalkiness in the endosperm. TEM analysis provided novel evidence that numerous vacuole-like structures remained among loosely packed starch granules in the inner endosperm cells upon exposure to the dry wind in the middle grain-filling stage (Fig. 5). Time course analysis of absolute qPCR in each tissue clearly indicated that starch biosynthesis-related genes in the inner chalky zone were more highly expressed than in the outer zone regardless of treatment at the stage of development (Table S2), which agreed with the image analysis based on the microscopic observations (Fig. 4a and d). These data indicated that starch accumulation would have more actively occurred in the inner zone than the outer zone at the stage, consistent with the starch accumulation pattern generally accepted in rice endosperm (Hoshikawa 1967). Moreover, the gene expression analysis (Fig. 3) and microscopic observations (Fig. 4) strongly suggest that partial reduction in starch synthesis, but with no starch degradation, occurred in the inner zone upon osmotic adjustment during the dry wind treatment (Fig. 3, Table S2). Therefore, we concluded that the preservation of vacuolar structures in the cytosol accounts for dry wind-induced air space formation confined to the inner endosperm, resulting in ring-shaped chalkiness (Fig. 1c), as illustrated in Fig. 6.

It is well recognised that the presence of air spaces exhibited between loosely packed starch granules causes chalkiness (Tashiro and Wardlaw 1991). Several researchers have shown that during normal development, the packing density of amyloplasts increased as the volume occupied with other organelles decreased (Del Rosario et al. 1968; Hoshikawa 1989). This is what we observed in the control in this study (Figs. 4 and 5). To date, most investigators have been conducting microscopic observations, mostly focusing on the surface structure of starch granules using scanning electron microscopy in dried chalky kernels (Zakaria et al. 2002; Yamakawa et al. 2007; Wada et al. 2014; Xi et al. 2014; Kaneko et al. 2016; Dou et al. 2017; Nakata et al. 2017). Because the tissues need to be dehydrated prior to scanning electron microscopy observations under a vacuum, this approach does not provide any information on what exactly occurred in the cytosol in the chalky zone. It appears likely that TEM observations have rarely been used to study rice chalkiness because this approach is technically demanding in hard tissues such as mature kernels. In this work, adoption of the recent improvement of suitable fixation of rice seeds in TEM (Saito et al, 2010, 2012) allowed us to identify the source of air spaces formed along the chalky ring in dry wind-treated kernels. Microscopic observations suggest that the vacuole-like structures observed in the inner endosperm in dry wind treatment may be composed of vacuoles and protein storage vacuoles (Fig. 5g and h); although, this will require further analysis. Regarding another chalkiness, white-belly rice, it has been suggested that the incomplete accumulation of protein bodies may be one of the major causes of chalky formation, as well as insufficient starch accumulation (Xi et al. 2014). We have similarly noted that the preservation of numerous protein storage vacuoles lacking the storage proteins at osmotic adjustment is closely associated with the formation of heat-induced rice chalkiness, called white-back kernels (Wada et al., unpublished data). It is generally known that the accumulation of protein bodies in the inner chalky zone, which we targeted here, is much smaller than in outer endosperm (Ellis et al. 1987; Hoshikawa 1989), consistent with our observations (see Fig. 4b and e). Because vacuoles contain vacuolar sap composed of water and small molecules (Taiz and Zeiger 2010), it is reasonably assumed that the osmotically adjusted cells located in the inner zone would have higher water content than the outer zone prior to kernel dehydration. Higher water content has been detected in heat-induced chalky region by MRI in the middle stage (Ishimaru et al. 2009). It is also anticipated that these vacuolar structures, preserved among various sizes of starch granules, would either shrink by losing water or rupture during kernel dehydration. Our TEM observations during the kernel dehydration prompted us to speculate that tonoplast degradation might occur, leading to the breakdown of compartmentation in the osmotically adjusted cells. In an early study, Tashiro and Wardlaw (1991) documented that ‘premature autolysis’ of starch may occur in addition to disrupted starch synthesis. During the dehydration process, it is possible that reactive oxygen species may be involved in the breakdown process as a second messenger (Mittler et al. 2004; Skopelitis et al. 2006; Onda et al. 2009), which will need to be investigated further.

The work presented here confirmed the previous suggestion (Wada et al. 2014) that a temporary reduction in starch biosynthesis occurred in the inner tissues at low water potential, without starch degradation. In rice endosperm, the accumulation of starch begins from the innermost cells in the tissues, and it continues toward the cells of the peripheral part in the tissues to completion (Juliano 1985; Hoshikawa 1989), suggesting that there is a substantial lag time on the starch accumulation rate between inner and outer zones. Absolute qPCR analysis (Table S2) and the image analysis in light microscopy (Fig. 4) revealed that starch biosynthesis in the inner endosperm more actively occurred than in the outer endosperm at the stage examined, implying the presence of the lag on the starch accumulation across the endosperm according to the starch accumulation pattern (Juliano 1985; Hoshikawa 1989). Because the observed spatiotemporal downregulation of most starch biosynthesis-related genes in the inner zone was tightly associated with changes in PWP (Figs. 2 and 3), shoot water deficit could alter starch biosynthesis only temporarily in the inner zone. In the outer zone, there were several genes similarly downregulated at low PWP, although normal amyloplast development occurred with no formation of vacuole-like structures (Fig. 5i), showing no treatment difference in the gap spaces in the outer zone (Fig. 4f), contrastingly different from the inner zone (see Fig. 5e). Based on these observations, it is reasonably interpreted that the temporal downregulation observed in the outer zone was not sufficient enough to structurally alter amyloplast development to cause chalkiness. In this view, it is likely that a spatiotemporal disruption of starch biosynthesis occurred mainly during the midpoint of starch accumulation, consistent with the time of appearance of MWR (Nagato and Kobayashi 1959).

It has been recognised that the quantity of RNA in eukaryotic cells is in the range of 10–100 pg per cell (Alberts et al. 2002; Nakazono et al. 2003). Among the genes analysed, the expression levels of most starch biosynthesis-related genes were substantially high, although this was not necessarily true for other genes (Table S2). For *AGPL2*, 16 log_2_ copies ng RNA^−1^, corresponding to 650–6500 copies cell^−1^, were detected in the inner zone, whereas the expression level of α-amylase-related genes including *Amy3A* was < 2 copies cell^−1^. Because the expression of 1–10 copies cell^−1^ is biologically insignificant (Gambetta et al. 2013), our data suggested that the expression level of the potential candidates, α-amylase genes, can be negligibly low at low water potential (Table S2), consistent with the findings of previous work using prefixed ^13^C analysis (Wada et al. 2014). Another interpretation is that the expression was too low to detect since the expression occurred in a much smaller fraction in the sampled tissues. However, this may not be the case because the volumetric ratio of chalky area to the inner tissue volume sampled was quite large, reaching a mean of 65% (*n *= 6) of the tissue sampled as ‘inner tissue’. Therefore, it is postulated that the sampled tissue segment can largely be regarded as the expression of genes expressed at the chalky ring area, excluding the outer tissue, where starch synthesis was suggested to occur slowly, compared with that in the inner zone (Table S2). If α-amylase activity occurred, the typical structure would be observed on the surface of starch, although this was not observed previously (Wada et al. 2014). Taking these findings together, α-amylase activity is unlikely to be involved in dry wind-induced chalky formation.

It is not surprising that a relatively small portion of air space could cause significant loss of transparency in chalky cells that occurred in a part of rice endosperm, as suggested previously (Wada et al. 2014). In this work, the ratio of the *gap* space observed in the chalky zone was estimated to be 18% of the chalky cells between the third and fifth layers, counting from the central point. It was previously reported that the size heterogeneity of starch granules was large due to the reduction in starch biosynthesis in the inner zone (Wada et al. 2014). This in turn means that the volume of cytosol (i.e. the *gap* space) should be large. Therefore, it may be premature to conclude that chalky formation is attributable only to the surface geometry of starch granules. Even though there was no significant treatment difference in the percentage of *gap* space in the central area, it is not unrealistic that the same mechanism exists in the central area, when considering that the corresponding region exhibited a slight loss of transparency due to the presence of few vacuole-like structures, as demonstrated microscopically (Fig. 5g).

In general, both V-ATPase and V-PPase play an essential role in regulating the vacuolar volume (Maeshima 2001; Taiz and Zeiger 2010). Li et al. (2014) reported that the expression of V-PPase was correlated with genetically formed chalkiness. We showed that the expression of the V-PPase-encoding gene was much higher than that of the V-ATPase-encoding gene in rice endosperm developing at the stage of development (Table S2), agreeing with general observations in young tissues (Maeshima 2001). However, no clear upregulation of these vacuolar proton pump-related genes was unexpectedly observed during the duration examined. It is also known that vacuoles containing hydrolytic enzymes are involved in vacuolar autophagy during drought (Marty 1999; Bassham et al. 2006). Autophagy has been shown to function in organelle degradation, such as that of chloroplasts and plastids (Wada et al. 2009; Izumi et al. 2015). In recent studies using mutants with knockout of autophagy-related genes, *ATG4b* and *ATG7*, the autophagy system was shown to function in the degradation of chloroplasts in the leaf and plastids in the root, respectively (Wada et al. 2009; Izumi et al. 2015). Analysis of these genes showed that the expression levels were relatively low, ranging between 8 and 9 log_2_ copies ng RNA^−1^. When considering these data, it seems unlikely that these autophagy-related genes, as well as the vacuolar proton pump-related genes, are directly involved in the observed vacuolar preservation at least up to 72 h after the treatment we applied.

Kobata et al. (2004) reported that MWR increases under high temperatures plus low light intensity, but decreases when spikelet numbers are reduced by thinning, which implies that the source ability per grain affects the number of MWR. Under 24-h dry wind conditions, a transient increase in ^13^C-labelled assimilates in kernels from mature organs was suggested to contribute to kernel development (Wada et al. 2014). In contrast, when the duration was prolonged to 48 h, endosperm cell expansion was partially inhibited, as the filled kernels and the kernel weight both slightly declined (Fig. 1a, b). As the occurrence of chalky rice caused under heat conditions was often attributed to a lack of assimilates in the endosperm (Kobata et al. 2004), this could be due to a loss of assimilate input. However, it should not be overlooked that a reduction in the weight of perfect rice, harvested at the same within-panicle position (presumably the same developmental age), was observed under the 48-h dry wind treatment, as well as MWR (ring-shaped chalkiness) (Fig. 1b). Hence, it may be premature to conclude that a low assimilate supply to the kernels themselves is the direct cause of chalky formation. When dry wind conditions last for >48 h, it is anticipated that sugar starvation would occur in part of the endosperm, as similarly suggested by the simulation of prolonged drought (McDowell 2011). As discussed above, we did not find any evidence for the involvement of these vacuole-related genes in chalky formation at least during the duration analysed. However, if autophagy induced cell-specifically in the putative chalky zone, the cell osmotic potential should decline to maintain the vacuolar (and cell) volume by promoting the entry of water into the cells. It is also possible that vacuoles may engulf starch granules (Wang et al. 2013). If this holds true, then short-term dry wind treatment may be the precondition to promote autophagy or vacuolar pumps in the cells.

In conclusion, our data indicated that, together with a partial reduction in starch biosynthesis, the preservation of vacuole-like structures occurs as a consequence of osmotic adjustment in the inner endosperm during hot and dry wind-induced low water potential, as summarised in Fig. 6. These responses consistently observed in the inner endosperm are responsible for air space formation in MWR. We propose that chalkiness is a form of acclimation to such multiple environmental stresses. In addition, an increase in vacuolar components through osmotic adjustment may be a universal cellular event that precedes chalky formation under other stress conditions, such as low light intensity and heat stress. To test this hypothesis in the target rice endosperm cells, some cell-specific analysis will be required. A newly developed cell metabolome analysis (Nakashima et al. 2016) combined with time-course TEM observations under stress conditions may be a powerful methodology to further extend our understanding of rice chalky formation at cell level.

### *Author contribution statement*

CM-K and HW conceived the study. YH, CM-K, and HW performed the experiments, analysed data, and prepared the manuscript. HN, SM, KH, YO, TN, and HN participated in analysis of data and assisted in writing the manuscript. All authors read and approved the manuscript.

## Electronic supplementary material

Below is the link to the electronic supplementary material.

Supplementary material 1 (DOC 950 kb)

Supplementary material 2 (DOC 2036 kb)

Supplementary material 3 (DOC 167 kb)

Supplementary material 4 (XLSX 29 kb)

## Abbreviations

DAH: Days after heading
MWR: Milky-white rice
PWP: Panicle water potential
