# Supplementary material for: Evidence for preservation of vacuolar compartments during foehn-induced chalky ring formation of Oryza sativa L
Source: Planta. 2018 Aug 11;248(5):1263–75. doi: 10.1007/s00425-018-2975-x (PMC6182326; doi:10.1007/s00425-018-2975-x)
Supplement: Supplementary file 1 — Supplementary material 1 (DOC 950 kb) [file 425_2018_2975_MOESM1_ESM.doc]

Figure S1.


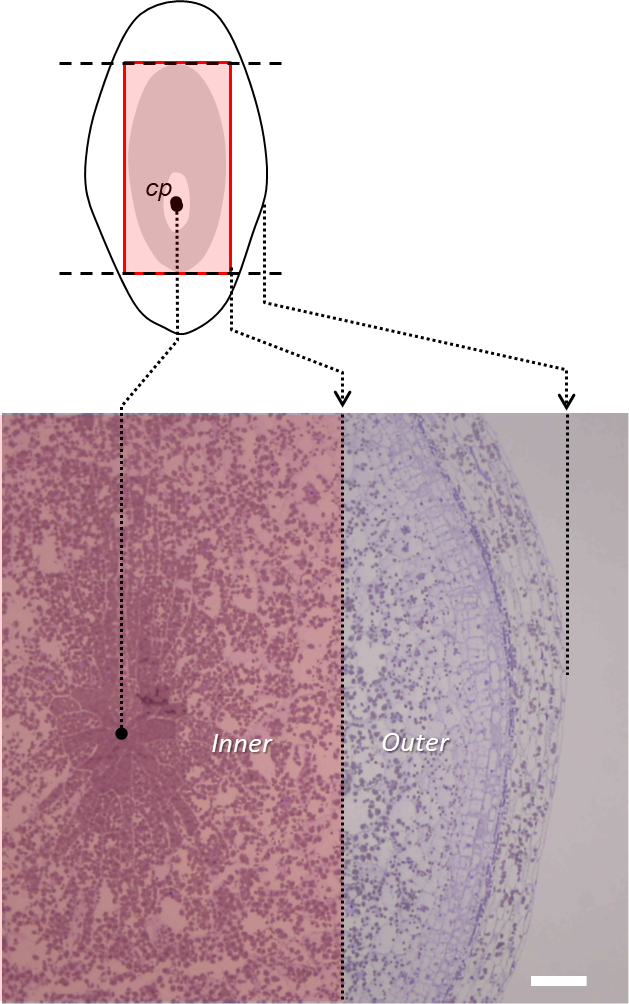


Fig. S1. Schematic diagram of the transverse section of developing kernel at 14DAH. After tissue extraction with the Biopsy punch along the dashed lines, ‘inner’ (shown in the red rectangle) and ‘outer’ tissues were individually sampled along the dotted line (see the image of the section) (see Materials and methods). ‘*cp*’ indicates the central point of endosperm. The gray zone corresponds to the putative chalky zone, where the starch accumulation actively occurs at the stage. The bar indicates 100μm.
