# Supplementary material for: Evidence for preservation of vacuolar compartments during foehn-induced chalky ring formation of Oryza sativa L
Source: Planta. 2018 Aug 11;248(5):1263–75. doi: 10.1007/s00425-018-2975-x (PMC6182326; doi:10.1007/s00425-018-2975-x)
Supplement: Supplementary file 2 — Supplementary material 2 (DOC 2036 kb) [file 425_2018_2975_MOESM2_ESM.doc]

Figure S2.


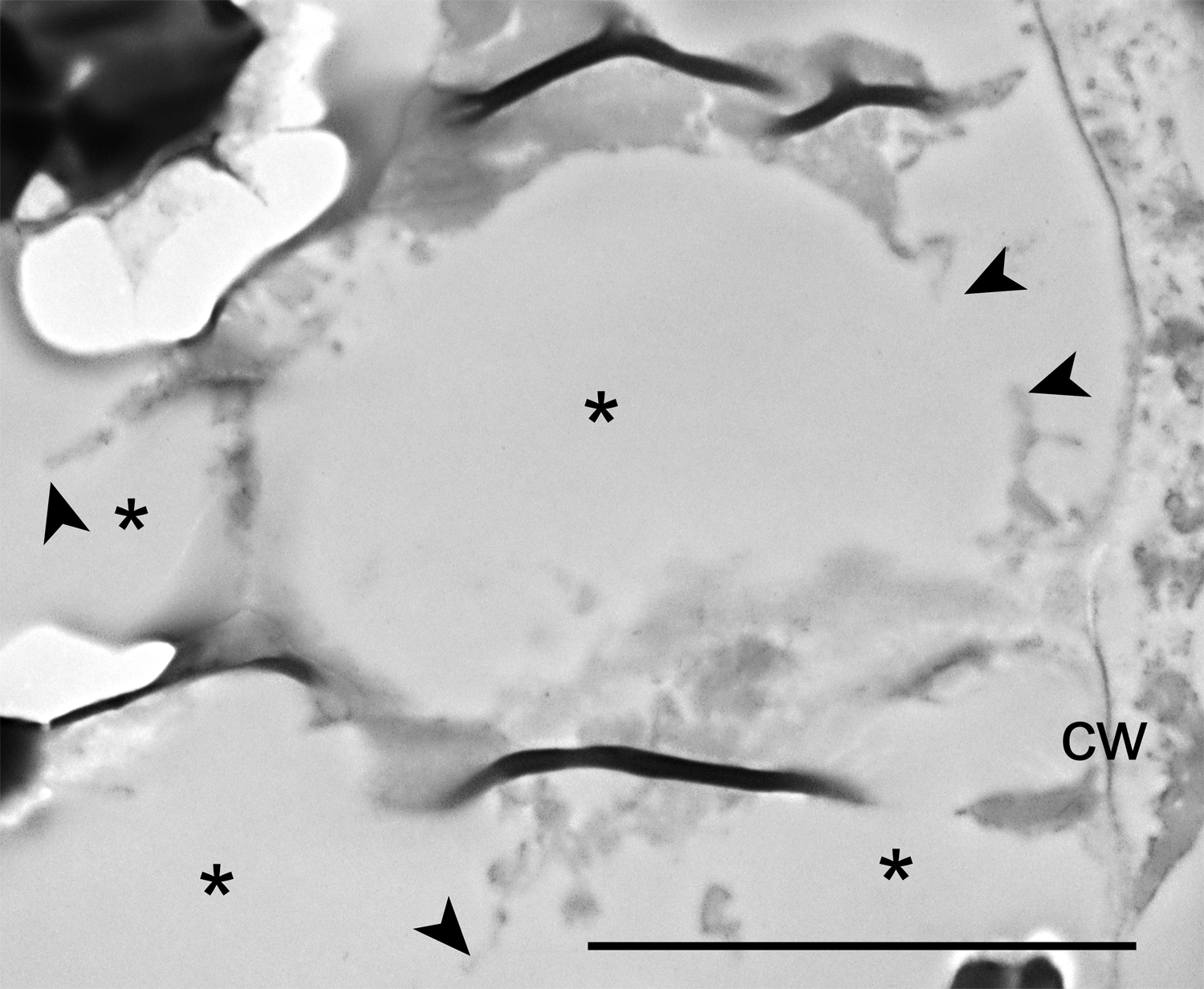


Fig. S2. A TEM image of the chalky zone, corresponding to the inner endosperm cells in dry wind treatment during kernel dehydration. Note that tonoplast degradation is presumed to occur in the cells, resulting in the compartmentation breakdown. ‘CW’ indicates cell wall. Each asterisk and arrowhead indicates vacuole-like structures remained in the cells and tonoplast presumably broken down, respectively. The bar indicates 5 μm.
