# Supplementary material for: Evidence for preservation of vacuolar compartments during foehn-induced chalky ring formation of Oryza sativa L
Source: Planta. 2018 Aug 11;248(5):1263–75. doi: 10.1007/s00425-018-2975-x (PMC6182326; doi:10.1007/s00425-018-2975-x)
Supplement: Supplementary file 3 — Supplementary material 3 (DOC 167 kb) [file 425_2018_2975_MOESM3_ESM.doc]

Table S1.

| Table S1. Primer sequences used in quantitative RT-PCR. | | | | |
| --- | --- | --- | --- | --- |
| Gene name | Description | Locus ID | Forward primer (5'-3') | Reverse primer (5'-3') |
| *AGPS1* | ADP-glucose pyrophosphorylase small subunit 1 | Os09g0298200 | ACGCCTTAATCCCTAGCGGAAC | TGCTGCAAGGCCCAACCTTATG |
| *AGPS2b* | ADP-glucose pyrophosphorylase small subunit 2b | Os08g0345800 | CAACAATCGAAGCGCGAGAAAG | CAATCTAGTCCCTGCACCAC |
| *AGPL1* | ADP-glucose pyrophosphorylase large subunit 1 | Os05g0580000 | CATGTGCTCCTGTTGGAGAGAGTC | TGAATTACACGGCCTGAACTGTCG |
| *AGPL2* | ADP-glucose pyrophosphorylase large subunit 2 | Os01g0633100 | TACTGGAACTGCACGATGTGTG | GGGAGGATTGTGTCCGAAGATGTG |
| *Amy3A* | α-Amylase 3A | Os09g0457400 | GAGGGTCATCACCAAGATCG | TGTGTAGCTAGCTTGCGAGC |
| *ATG4b* | Autophagy-related gene 4b | Os04g0682000 | TGGAACATCAACCTACATTGCTG | TGCAGTGGTATGAAGAAGTGTCTG |
| *ATG7* | Autophagy-related gene 7 | Os06g0614900 | GCCTGGATTGTCCTCCATTAC | GATGCGGCAATAGACCAAGTG |
| *BE1* | Branching enzyme 1 | Os06g0726400 | TTGGTGGCCATGGAAGAGTTGG | TTCTGGTACTCCTGGCATTCCC |
| *BE2b* | Branching enzyme 2b | Os02g0528200 | TTTGGCAGGATCCATCACAC | TGGTGTTCTCATTCCGCTGG |
| *BT1-2* | ADP-glucose transporter 1-2 | Os02g0202400 | AGGTTGCGTGAAGTGGTTTGGG | TGCTTCCATCATCACACCCATGC |
| *CIN2* | Cell wall invertase 2 | Os04g0413500 | TTCTCAAGGACAGGGTGGTCAAGC | TCAGCCTGTGCAGTTTGTAGCC |
| *CIN7* | Cell wall invertase 7 | Os09g0255000 | TGTGCACCGACTTGACAAAGTCG | GCGATATGGTTTTGTGATCGTCG |
| *GBSS1* | Granule-bound starch synthase 1 | Os06g0133000 | TCCGTCATTCCTGGAGAAGGTTTG | TCAACTCCAGTGTCAGGTCCGTAG |
| *ISA1* | Isoamylase1 | Os08g0520900 | TGACTTGCAACGGTTCTGCTCTC | GCCAAGGGACTCGCATTGTTTG |
| *NIN8* | Alkaline/neutral invertase 8 | Os02g0550600 | TAGAGGAGGATAAGGCAATGAAGC | AGTCAAACGGAGGGATGAGCAG |
| *PUL* | Pullulanase | Os04g0164900 | TGGGACTTTGGTGAGGTTGCAC | CGCGGATCCTATCGTTGAAACTAC |
| *SUT1* | Sucrose transporter 1 | Os03g0170900 | TGCCTCGTCCTCTTTGCGTTTC | GCGATGACCACCTGAGGGATG |
| *SUT2* | Sucrose transporter 2 | Os12g0641400 | ACCATATGCAATGGCTGCTAGTCG | ACCCAGTGACACAATAACCTGTGG |
| *SuSy2* | Sucrose synthase 2 | Os06g0194900 | TCGGAGTTCAACCACAGGTTCC | ATGGTGTCAAGCACACGCTTTG |
| *SuSy3* | Sucrose synthase 3 | Os07g0616800 | AGCTGAGTGTCCCTGAATACTTGC | GCACAAAGTTGTTCTGGGTGCTTC |
| *SS1* | Starch synthase 1 | Os06g0160700 | TATAAGGCCCGATGTGCCTCTG | GTCACCAGATCCAAGCATGACG |
| *SS2a* | Starch synthase 2a | Os06g0229800 | TCATTGACGCTCCTCTCTTCCG | TCATGCGCTTCATGATTTCCTGTC |
| *SS3a* | Starch synthase 3a | Os08g0191500 | CGACGTAGACCATGACAAAGAC | CAAACCAAGAAGAGATTGCTCTG |
| *TMT1* | Tonoplast monosaccharide transporter 1 | Os10g0539900 | GTCCAAAATGGGCAGACTTATTC | GCAAGAAGAACACCAACACCAG |
| *VHA-A* | Vacuolar H^+^-ATPase subunit A | Os06g0662000 | TGTGGAGCGTGCAGCTAATG | TTTTGCGACTAGGACATCTTCAC |
| *VHP1;3* | Vacuolar H^+^-pyrophosphatase 1;3 | Os02g0802500 | AGGACGGACGGAGATTTTCG | CGTAGAGATAGTGTCCCACTACCAC |
| *VHP1;5* | Vacuolar H^+^-pyrophosphatase 1;5 | Os05g0156900 | GCATCGTCTTCAACCACCTCTG | TCTTCTTGTTTATCCATGCGTGC |
| *eEF1a-1* | Eucaryotic elongation factor 1α-1 | Os03g0177400 | TCAAGTGGTGCTTTCCATATCTG | GCAACAACTGAAATGACCGTCG |
